# A facile and general route to synthesize silica-coated SERS tags with the enhanced signal intensity

**DOI:** 10.1038/srep14934

**Published:** 2015-10-09

**Authors:** Youlin Zhang, Xiaokun Li, Bin Xue, Xianggui Kong, Xiaomin Liu, Langping Tu, Yulei Chang

**Affiliations:** 1 State Key Laboratory of Luminescence and Applications. Changchun Institute of Optics, Fine Mechanics and Physics, Chinese Academy of Sciences. 130033, Changchun, China

## Abstract

Silica-coated SERS tags have been attracting greater attention in recent years. However, the reported methods to synthesize these tags are tedious, and often subjected to the limited signal intensity. Here, we report a facile and general method to prepare the silica-coated Ag SERS tags with the enhanced signal intensity by no introducing the primers. This approach mainly depends on the colloidal stability of the Ag NPs in alcohol solution. By decreasing the concentration of salt in Ag NP solution, the citrate-stabilized Ag NPs can be well dispersed in alcohol solution. Based on this, the Ag SERS tags can be directly coated with thickness-controlled and homogeneous silica shells. This approach is highly reproducible for silica shell growth and signal intensity, not depending on the properties of Raman molecules, proved by 7 kinds of the Raman molecules. Moreover, this kind of SERS tags coated with silica hold the stronger SERS signals than the traditional method due to no interference from the priming molecules.

Surface-enhanced Raman scattering (SERS) is a highly-sensitive vibrational spectroscopy technique to detect analytes on or near the surface of plasmon nanostructures, greatly expanding the role of Raman spectroscopy^1^. In the present, this technique has been employed to construct new nanoprobes (named as SERS nano-tags) that merge noble metallic nanoparticles (NPs) with Raman molecules^2^. Such SERS tags possess strong Raman signals from the Raman molecules and can be employed to inderectly recognize the target molecules, presenting labeling functions same with the fluorophores such as organic dyes and quantum dots (QDs)^2^,^3^,^4^,^5^,^6^,^7^,^8^,^9^,^10^,^11^,^12^,^13^. Moreover, compared to other tags, *e.g.* QDs, and fluorescent dyes, SERS tags are superior in multiplexing, ultra-sensitivity, high-photostability, and quantitative abilities^2^,^13^,^14^,^15^. Importantly, as NIR optical probe, SERS nano-tags overcome the shortcomings of the other NIR probes, *e.g.* no photobleaching and the much more number of distinct signals in the NIR window. Therefore, SERS nano-tags are under more and more active investigation for *in vivo* applications^7^,^8^,^9^,^10^.

In SERS nano-tags, Ag NPs have been proven to be the best substrates for SERS analysis. Among them, citrate-capping Ag NPs are expected to be an ideal candidate for SERS studies, because citrate groups as the much weaker capping agents on the NP surface, are easily exchanged with Raman reporter molecules to ensure the intimate contact between the Raman moleules and Ag NPs^16^. However, such SERS tags are unstable under the ambient conditions, oxidation in the air, aggregation in salt solution and dissociation of Raman molecules, particularly, biotoxicity to limit their applications^2^,^17^. For their applications, a general approach is to coat a silica shell to overcome the above shortcomings. In addition, the shell can also bring the following merits: (1) hinders the Ag NPs from the medium that could produce the noise; (2) provides a convenient functionalized surface for coupling the other molecules^2^.

Encapsulating silica on the NPs suface demands that the NPs are stable in alcohol solution before the hydrolysis/condensation of tetraethyl orthosilicate (TEOS). Therefore, prior to encapsulation of silica, a new interface has to be introduced by using some priming molecules to modify the NP surface in order to obtain the colloidal stabilization of the NPs in alcohol solution (See Fig. 1A). This demand introduces much more complication into the coating process. Worse still, introducing the priming molecules will occupy a portion of sites of the Raman molecules on the NP surface, which give rise to the other shorcomings: (1) the reduce of the reproducibility and intensity of signal from the SERS tags^17^,^18^,^19^; (2) unable to obtain silica-coating SERS tags for some Raman molecules that hold the weaker affinity to the NPs than the priming molecules^2^. The above drawbacks are, as you know, highly prejudicial for bioapplication or multiplex high-throughput screening.

Schlucker methods describe the LBL deposition or terminal SiO_2_ precursors strageties to obtain the reproducible and maximum signal^4^,^20^. In contrast to most of the reported procedures, the improvement is at the cost of preparation time, the more chemicals, and the harder process to handle. Moreover, it is still difficult to obtain SERS tags coated with silica for some Raman molecules. Therefore, developing a more straightforward, facile, and reproducible approach to synthesize silica-coated SERS tags is very demanding.

In this work, we have developed a facile and universal method for preparing silica-coated SERS tags by no introducing the priming molecules. Our results demonstrated the citrate-stabilized Ag NPs could be well dispersed in alcohol solution by decreasing the concentration of salt in solution and the citrate groups on the surface of Ag NPs could promote the silica growth. Based on this, the new synthesis route of silica-encapsulated SERS tags was designed and schematically illustrated in Fig. 1B. The key of this method was to decrease the amount of the residual salt in the Ag NP solution before encapsulating the SERS tags with silica. We found it very effective in fabrication of silica-coated core-shell SERS tags. Furthermore, the intensity of silica-coated SERS tags was stronger than that of traditional method because the SERS reporters could form a self-assembled monolayer (SAM) on NP surface due to no interference from the priming molecules.

This versatile strategy was fit for all the Raman molecules with affinity to Ag NP, not depending on its chemical nature, as proved here by selecting 7 kinds of Raman molecules. The silica growth depended on the Ag NPs, nor the Raman molecules adsorbed on the Ag NPs.

## Results and Discussion

**Figure 1:**
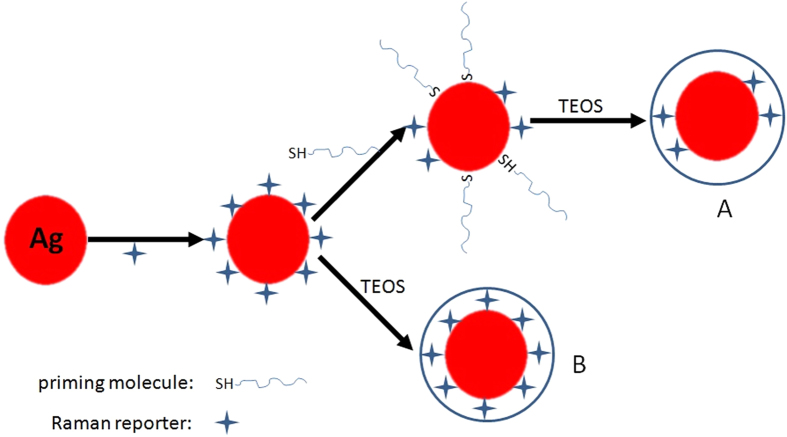
Schemetic illustration of the preparation of the silica-coated core-shell SERS tags: (A) the reported approach based on the priming molecules, (B) the developing approach for directly coating Ag SERS tags with silica.

In silica-coating Ag NP process, the colloidal stabilization of the Ag NPs in alcohol solution is the key to realize silica encapsulation. However, various reports have demonstrated alcohol can cause the aggregation of metal NPs, preventing the formation of silica-coated single metal NPs^21^,^22^,^23^,^24^. Therefore, the silica-coating methods have no choice but to introduce the priming molecules to provide the colloidal stability in alcohol solution and promote the silica growth (Fig. 1A). For realizing our strategy (Fig. 1B), we must firstly solve the problem about the stability of citrate-stabilized Ag NPs in alcohol solution.

According to DLVO theory, there are many potential factors that can affect the stable status of Ag NPs^25^,^26^. Colloidal stability is subject to a balance between the electrostatic repulsion (*V*_*elec*_) and van der Waals attraction (*V*_*vdW*_).


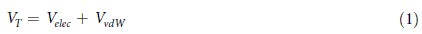


where *V*_*T*_ represents the total interaction potential. The *V*_*elec*_ is the dominant force at the transition to the aggregation state and simply expressed as following^27^:


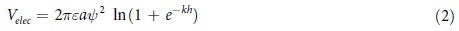


where *ε* denotes the dielectric constant of the medium, *ψ* is the surface potential, *h* is the gap between the surfaces of the colloids and *k* is the inverse of the Debye length,


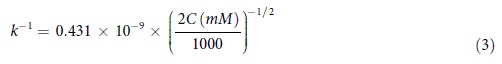


where *C* is the concentration of salt in the solution.

Polar organic solvents with a smaller *ε*, such as alcohol and acetone, *et al.*, are added in the citrate-capped Ag NPs aqueous solution, resulting in the decrease of *ε* of solution. According to Equation (2), a decrease in *ε* can result in a decrease of *V*_elec_, which explains why the Ag NPs are not stable in alcoholic solution. Similarly, according to Equation (3), the increase of salt concentration also leads to the aggregation of Ag NPs in aqueous solution. Presently, the role of salt on Ag NP aggregation is a general common sense^28^. For example, metal cation-induced NP aggregation has been applied to construct nanostructure materials, and develop different detection schemes^29^,^30^.

In silica-coating process, we think the addition of alcohol leads to the aggregation of Ag NPs due to the decrease of *ε* of alcohol (such as methyl alcohol, 32.7; ethanol, 24.5; isopropanol, 18.3), comparing with water (*ε*, 80). However, Equations (2) and (3) also indicate that decrease of the concentration of salt can increase the *V*_*elec*_ of Ag NPs, which might improve the stability of Ag NPs in alcoholic solutions. In the following work, we will discuss in detail the stability of Ag NPs in alcoholic solutions through varying the concentration of salt in the synthesized Ag NP solutions in order to develop a reproducible and simple method for coating SERS tags.

### The stability of Ag NPs in alcoholic solutions

**Figure 2:**
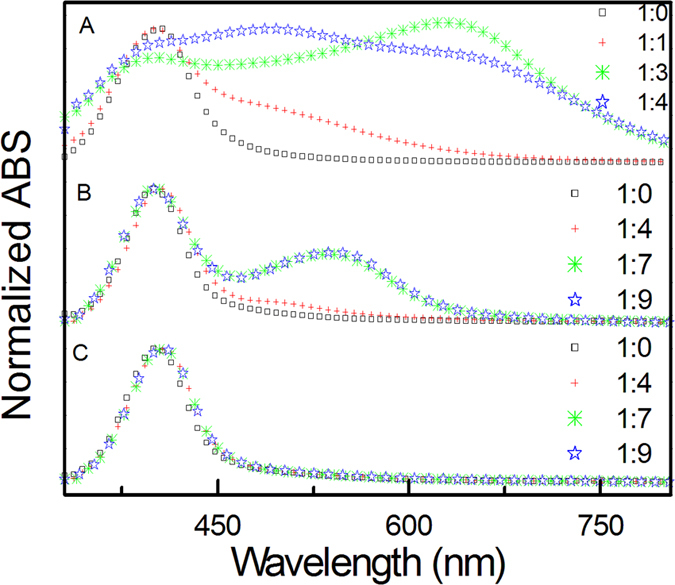
Normalized optical absorption spectra with different ratio of water to isopropanol of Sample 1 (A), Sample 2 (B), and Sample 3 (C). Volume ratios between water and isopropanol are given in the figure.

UV-visible extinction spectrum is a valid approach to research the aggregated status of NPs in solution^31^. We studied the effect of salt on the stability of Ag NPs in alcoholic solutions in the following way: 1.0 mL Ag NP solution (Sample 1) was centrifugated after synthesis to remove as much as possible the supernatant. The residual Ag NP precipitation (ca. 10 μL) was redispersed in 1 mL water (Sample 2). Sample 2 was centrifuged again and the precipitation (10 μL) was dissolved in 1 mL water (Sample 3). In Fig. 2, the spectra of the Sample 1 present an extinction band maximized at 400 nm. When increasing the ratio of isopropanol to water from 0 to 4, the absorption spectra changes significantly (See Fig. 2A). A new band from 500 to 800 nm appears, shifting to longer wavelength. This is because aggregated NPs appear, as described by Mie theory^32^. When the isopropanol to water ratio was over 7:1, the Ag NPs were completely aggregated to the bottom of solution. Figure 2B,C show the absorption spectra of Samples 2 and 3 dispersed in solution of different isopropanol concentrations. As shown in Fig. 2B, when the ratio increases from 0 to 4, the absorption spectra remain basically the same. Obvious change occurs when the ratio goes up to 7 and 9, where a new band from 500 to 600 nm appears. But the shift is far less than that of Sample 1. As shown in Fig. 2C, with increasing the ratio from 0 to 9, no change in the absorption spectra is observed. These results indicate that centrifugation can vary the disperse ability of Ag NPs in alcoholic solution. The above experiments prove the removal of the original solution of the Ag NPs can increase the stability of Ag NPs in alcoholic solutions. To further illustrate the key factors to influence the stability of Ag NPs, we checked Sample 2 and 3 obtained by dialysis. In which the residual salt was diluted by 100 and 10000 times, respectively. The corresponding UV-vis absorption spectra in isopropanol-water mixture solutions behaved the same as aforementioned sample 2 and 3. The above experiments confirm the remnant is important in the aggregation of Ag NPs in alcoholic solutions.

**Figure 3:**
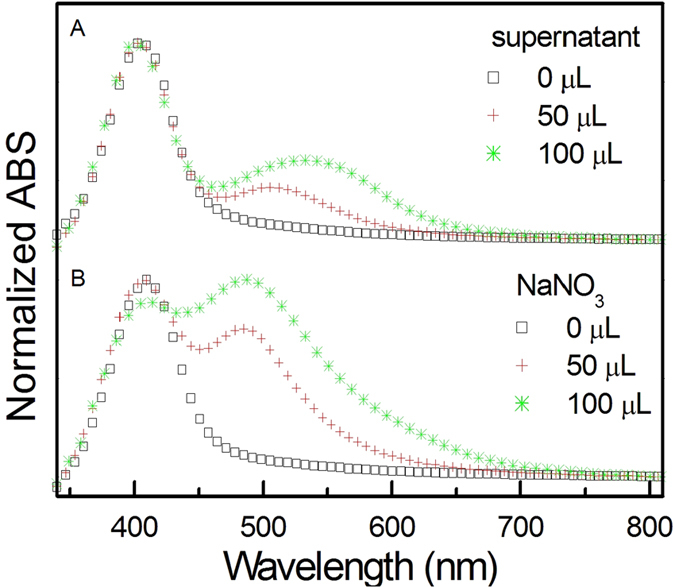
Normalized optical absorption spectra of Sample 3 in isopropanol solution with isopropanol to water ratio of 9 with addition of (A) different volumes of the supernatant of the Sample 1; (B) different volumes of the NaNO_3_ solution.

Another validating experiment was done by injecting different volumes of additional supernatant from Sample 1 into Sample 3 in alcoholic solution with isopropanol to water ratio of 9. As shown in Fig. 3A, with increasing the amount of supernatant from 0 to 100 μL, a new band between 500 and 600 nm appears, gradually shifting to longer wavelength. The supernatant primarily contains sodium ions and nitrate ions. Thus it is likely that sodium nitrate could contribute to the aggregation of Ag NPs. The NaNO_3_ solution (1.0 mM) was used to replace the supernatant, and the aggregation appeared in the absorptive spectra shown in Fig. 3B. Therefore it is confirmed that the residual salt in combination with isopropanol, rather than isopropanol itself, induces the aggregation of Ag NPs in alcoholic solution. In the absence of salt, Ag NPs are well dispersed in alcohol solution.

According to Equation (1), the Ag NP stability depends on a balance between the electrostatic repulsion and van der Waals attraction. As long as the repulsive barrier is greater than 10 k_*B*_T, the Ag NPs will be stable in solution^33^. When the repulsive energy is greater than 10 k_*B*_T, the collisions due to Brownian motion can not overcome the barrier and the colloids will be stable in the solution. When the repulsive energy is reduced below 10 *k*_*B*_T, the aggregation will occur due to Brownian motion. A decrease of *ε* of the medium accompanies with the reduction of repulsive barrier, inducing aggregation of the Ag NPs in alcoholic solution when it is lower than 10 *k*_*B*_T. On the other hand, the *V*_*elec*_ also depends on the concentration of salt. Assuming the same *ε*, the lower concentration of salt, the stronger the repulsion becomes. Table S1 gives the stable conditions of Ag NPs in alcoholic solution, which indicates that reduction of salt concentration can increase the stability of Ag NPs when *ε* is low. At last, the Ag NPs can be well dissolved in alcoholic solutions by reduction of salt concentration.

The addition of the supernant or sodium nitrate results in a new absorption band, and the new band becomes gradually stronger with the concentration of the salt increasing. The new band (450–700 nm) is ascribed to the longitudinal electronic oscillation mode of linear colloidal aggregates^28^. At the same time, a much broader and red-shifted band of the supernatant appears, which is because of the components of the supernatant being much more than NaNO_3_. There are plenty of reports on the aggregation of metal NPs seriously depending on the components of the salt^28^,^34^.

### Fabrication of SERS tags covered with an inert silica shell

The Ag NPs can be stable in alcoholic solution by removal of the residual salt. In the following, we will demonstrate whether the Stöber method can directly form a silica shell on the citrate-stabilized Ag NPs without priming molecules (See Supplementary information). The results indicate a silica shell can be directly grown on the surface of citrate-stabilized Ag NPs, confirming that the citrate groups on the surface of Ag NPs can serve as the active site to promote the silica growth.

These results portend silica-encapsulated SERS tags without the help of the priming molecules is also possible. The whole procedure of fabrication of silica-encapsulated SERS tags is illustrated in Scheme S1. Removing the residual salt in the Ag NP solution is also the key factor of this method. Firstly, the Ag NPs is centrifugated once, and then dispersed in the same volume of deionized water. The aim of this step is that the residual salt is diluted to 100 times, and also easily to couple the Raman molecule to the surface of Ag NPs. Secondly, the Raman molecule in ethanol solution is added into the above Ag NPs solution, reacting for 12 h, followed by the centrifugation of the mixed solution once and dispersed in the same volume of deionized water to remove the excessive Raman molecules. The above strategy results in SERS tags without the superfluous salt. At the end, the SERS tags are coated with silica, following the same procedure of coating the Ag NPs with silica.

**Figure 4:**
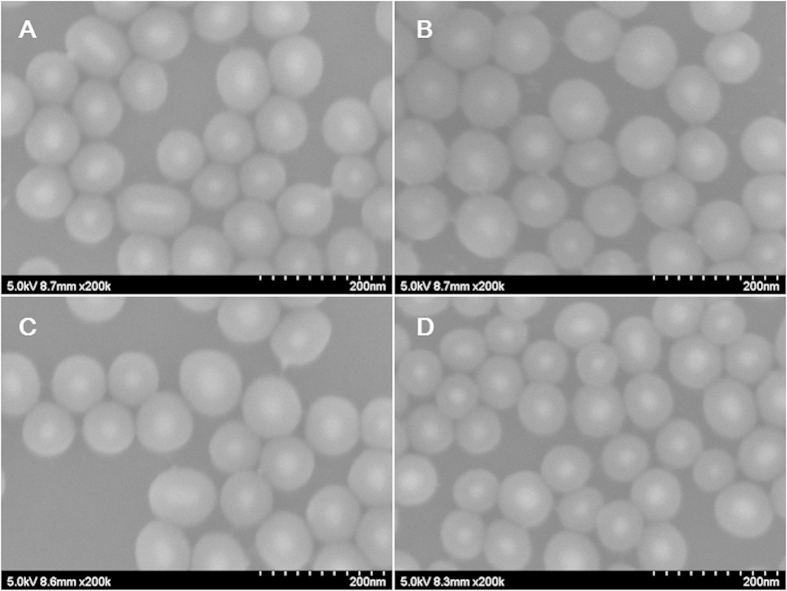
FE-SEM of silica-coated SERS-tags with different Raman reporters: (A) 4-MBA, (B) DTNB, (C) R6G, and (D) 4-MBA and DTNB.

Here, we firstly selected three kinds of typical molecules as Raman reporters to illustrate the feasibility of our approach. 4-MBA, DTNB and R6G were employed as the Raman molecules due to the fixed spectral recognization and good Raman scattering cross^2^. In addition, for 4-MBA and DTNB, their thiol groups hold a strong affinity to the Ag NP surface. R6G, however, has weak affinity to the Ag NP surface. We designed four kinds of SERS tags with reporters of 4-MBA, DTNB, R6G, and the mixed layer of 4-MBA and DTNB, respectively, to check the feasibility of the method. Figure 4 shows FE-SEM images of the silica-coated SERS tags. The results show the silica shells are homogeneous with thickness of about 25 nm. At the same time, Fig. 4 also indicates that the influence of Raman reporter molecules on the silica encapsulation is very weak.

**Figure 5:**
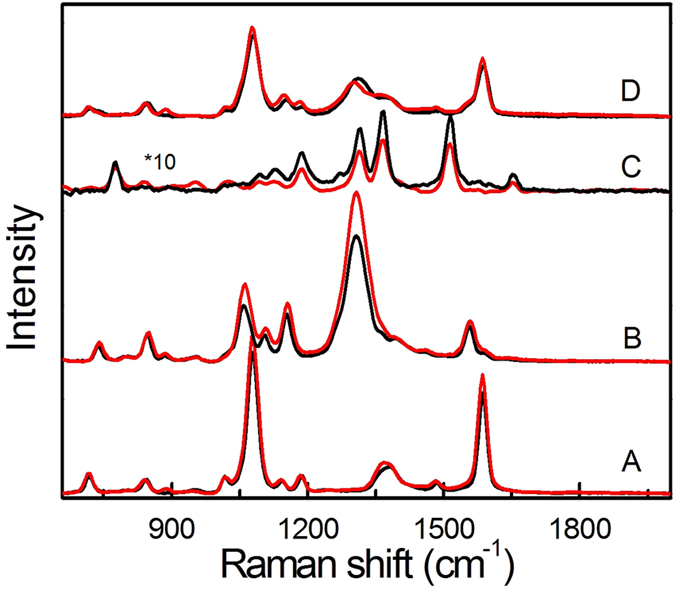
SERS spectra of aqueous suspension of SERS tags before (black line) and after (red line) silica encapsulated with different Raman reporters: (A) 4-MBA, (B) DTNB, (C) R6G, and (D) 4-MBA and DTNB. For R6G, the intensity is amplified 10 times. Both spectra were obtained with NIR laser excitation (785 nm).

We now turned to the influence of silica-encapsulation on the SERS signal. Figure 5 shows Raman spectra of silica-coated SERS tags and SERS tags before silica encapsulation. In Fig. 5, the SERS tags display strong and unique spectroscopic signatures. Obviously, the peak positions remain unchanged upon the encapsulation, indicating that the silica coating does not vary the structure of Raman molecules. The signal intensities of 4-MBA and DTNB change slightly after silica encapsulation, verifying no apparent loss during encapsulation due to the strong affinity to the Ag NP surface. The signal intensity of R6G is apparently weaker after silica encapsulation, indicating the loss of R6G during encapsulation, which could be due to that R6G holds weak affinity to the Ag NP surface. The enhancement factor of R6G on Ag NPs upon 785 nm laser is far smaller than that of 4-MBA on Ag NPs, in line with previous report^35^. The enhancement factor of silica-coated SERS tags are calculated according to the analytical enhancement factors (AEF) and shown in Supplementary information. Figure S4 shows the influence of shell thickness on the absorptive spectra of Ag NPs coated with silica shell. The electric field enhancement should possess an analogous spectral reliance with the absorptive spectrum. This has been affirmed by the simulation of electric field of Ag and Ag@SiO_2_ core-shell structure (Figure S5).

**Figure 6:**
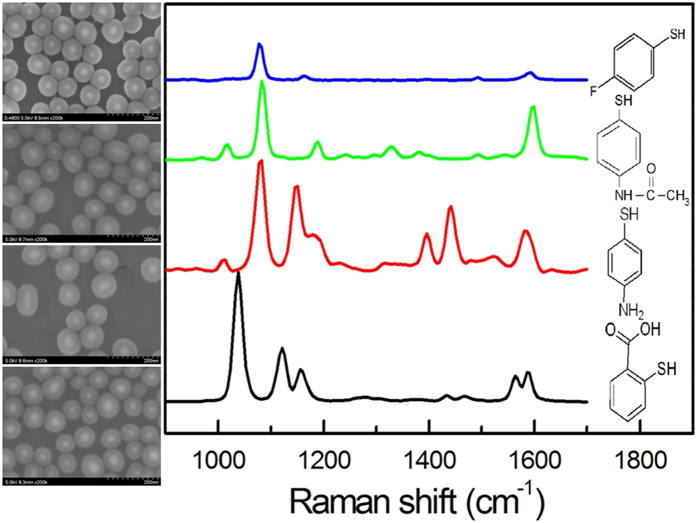
SERS spectra of 4 representative SERS tags. Left column: the SEM images of the corresponding SERS tags.

As previously reported, the –COOH groups possess chemical affinity to silica^36^,^37^. Therefore, some Raman molecules such as 4-MBA and DTNB can be acted as stabilizing agent, and in the following silica-coating process, the –COOH groups are employed as the active group to facilitate the hydrolysis/condensation of TEOS. So the described examples can not provide the sufficient evidences for the universality of our strategy. Figure 6 shows the SERS and SEM images of 4 representative SERS tags with their terminal groups of –F, –CH_3_, –NH_2_ or –H without the active site of –COOH groups. The results clearly demonstrate the versatility of our method, and indicate the groups to promote silica growth are not –COOH groups of the Raman molecules. Therefore, the citrate on the surface of Ag NPs is the active site to promote silica growth.

### Comparing our method with the traditional method

The signal intensity of the SERS tags is very necessary for the assay sensitivity.

**Figure 7:**
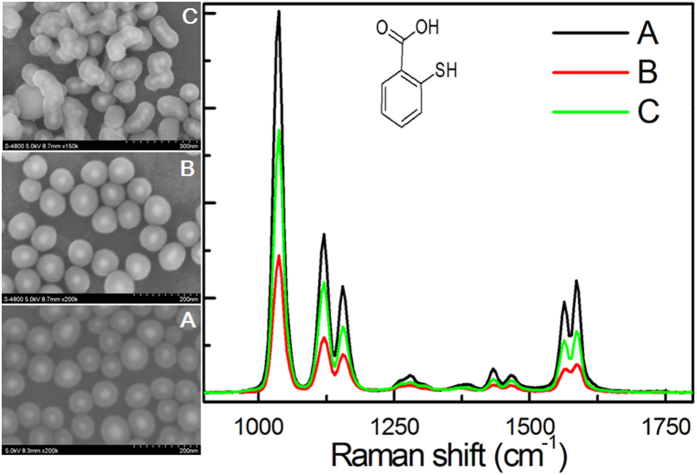
Influence of the surface coverage on the SERS signal strength: (A) complete SAM of 2-MBA compared to (B) a 8:1 of 2-MBA to MUA, and (C) a 40:1 of 2-MBA to MUA.

**Figure 8:**
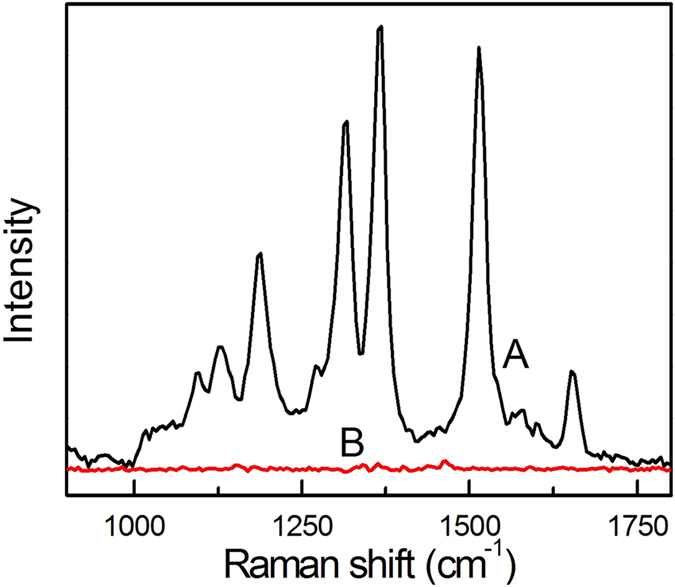
Influence of the surface coverage on the SERS signal strength: (A) complete SAM of R6G compared to (B) a 10:1 of R6G to MUA.

This requires that the Raman molecules form a SAM on the Ag NP surface in order to obtain the greatest amount of Raman molecules on the Ag surface. In our strategy, due to no introducing the priming molecules, a SAM of the Raman molecules on the Ag NP surface can be easily formed. Therefore, the signal intensity of silica-coated SERS tags with our method should be superior to the traditional method. In the following, we will provide the direct evidence. The previous articles have confirmed the MUA molecules could be acted as an effective stabilizing agent in alcohol solution and the –COOH groups of MUA could promote silica growth^36^,^37^. Recently, Nicolas’ group used the MUA as the priming molecule to develop a universal method to synthesize silica-coated SERS tags and thought their method was the best for the reported method with introducing the priming molecules^37^. Therefore, in order to present the advantages of our method, we also introduced the MUA molecules as the priming molecules to synthesize Ag@MUA/2-MBA@SiO_2_. The Ag NPs were firstly modified by the MUA for 1 h, and then the 2-MBA was added for another 3 h. The amount of 2-MBA increased besides referring to the reference^37^. The spectra and SEM of silica-coated SERS tags are shown in Fig. 7. The signal intensity of B is only 36% of A, which is similar with the reference^37^. In order to further increase the signal intensity of SERS tags, we further increase the ratio of 2-MBA to MUA with no changing the amount of MUA in the reaction. The signal intensity only slightly increases. When the ratio of 2-MBA to MUA is 40:1, the signal intensity can suddenly increase 1 fold (Fig. 7C). However, the SEM image indicates multiple Ag NPs are coated into a silica shell, proving the enhancement comes from the ‘hot spot’ effect caused by the aggregation of Ag NPs. This is maybe because the 2-MBA replaces the part of MUA on the surface of Ag NPs, reducing the stability of Ag NPs in silica-coated process. The above results demonstrate the obtained silica-coated SERS tags are far better than that from the traditional method. We also execute the same experiment for the R6G with the weak affinity to Ag NP. The results are shown in Fig. 8, and demonstrate that the Ag@MUA/R6G@SiO_2_ can not be obtained. All the results certificate that our method is superior to the traditional method with introducing the priming molecules. At the same time, compared to the Schlucker method, the coating process of our strategy is more simple, reproductive and general because of no additional treating process such as LBL deposition or the Raman molecule modification^4^,^20^. In addition, because our strategy also forms SAM of the Raman molecules on the surface of the Ag NPs, same with Schlucker method, the silica-coated SERS tags possess the same signal intensity.

Such SERS tags are inert to the interference of surrounding environment. For example, Ag@R6G@SiO_2_ were treated with 4-MBA, the Raman spectra showed only the R6G signature without any characteristic peak of 4-MBA (Figure S6). Stability study was also performed for the silica-coated SERS tags (shell thickness, 25 nm). High concentrations of salts cause irreversible aggregation of metallic NPs.^[46]^ The stability of Ag@SiO_2_ NPs was assessed according to the change of absorptive spectra in the absence and presence of high concentration salts (see Figure S7). The results demonstrate that Ag@SiO_2_ NPs are extremely stable in aqueous salt solution and no aggregation even after 12 months storage, and original SERS activity remains.

## Conclusions

In this work, a reproductive and general method has been developed for the preparation of Ag@SiO_2_ SERS tags by no introducing priming molecules. This method is subject to the concentration of salt in the solution. By decreasing the concentration of salt in Ag NP solution, the citrate-stabilized Ag NPs can be well dispersed in alcohol solution, which is well explained by the DLVO theory. In a following step, the carboxylic groups of citrate on the Ag NP surface is acted as active sites to promote the silica growth on the Ag NPs. This approach is demonstrated to be fit for different Raman molecules (7 Raman molecules) without affecting the SERS fingerprint of silica-coated SERS tags. Notably, the signal intensity of SERS tags prepared by our strategy is much higher than that of traditional method with introducing the MUA molecules as the priming molecules (the best method reported in reference)^37^. More importantly, the weak affinity ligand to Ag NPs can be also coated with silica due to no interference from the priming molecules. Compared to Schlucker method^4^,^20^, our stragety is very simple because silica can be directly coated on the surface of SERS tags.

## Experimental Section

### Materials

Tetraethyl orthosilicate (TEOS, 98%), and aminopropyltriethoxysilane (APTMS) were purchased from Sigma-Aldrich. 4-mercaptobenzoic acid (4-MBA), 5, 5’-dithiobis-(2-nitrobenzoic acid) (DTNB), Rhodamine 6G (R6G), thisosalicyilic acid (2-MBA), 4-aminothiophenol (4-ATP), 4-fluorothiophenol (4-FTP), 4-acetamidothiophenol (4-AMTP) and 11-mercaptoundecanoic acid (MUA) were bought from the Aladdin Industrial Inc. Sodium citrate, dimethylamine, anhydrous isopropanol, NaNO_3_, NaBH_4_ and NaCl were obtained from Beijing Chemical Plant. Silver nitrate (AgNO_3_, ≥99%) was purchased from Fluka. Deionized water was purified through a Milli-Q water purification system and the resistivity was 18.2 MΩ·cm.

### Preparation of Ag NPs

The preparation of Ag NPs followed our before article^36^. Simply, in aqueous solution, the AgNO_3_ was firstly mixed with sodium citrated, then, NaBH_4_ was rapidly added into the above solution. the concentrations of three reagents were 1 × 10^−3^ M, 7 × 10^−3^ M and 2 × 10^−7^ M, respectively. At last, the above solution was boiled for one hour, and the temperature was down naturally to room temperature.

### Stability in isopropanol

1.0 mL Ag NP solution (Sample 1) was centrifugated at 8500 rpm for 15 min, and the remnant (10 uL) was dispersed in 1 mL water (Sample 2). The obtained Ag NP solution was again centrifugated and the precipitate (10 μL) was redispersed in 1 mL water (Sample 3). The Sample1, 2 and 3 were dissolved in different isopropanol to water ratio solution with or no NaNO_3_.

### Synthesis of silica-coated Ag NPs

Sample 3 (2 mL) was added into isopropanol (8 mL) in a 15 mL plastic conical tube. Dimethylamine solution (0.2 mL, 30 wt.-%) was added to the above solution during stirring, and then the TEOS (0.6 mL, 10 mM) was added with three times within 6 h (at a time interval of 2 h). The following steps were described in our before article^29^.

### Preparation of SERS reporters-functionalized Ag NPs

4-MBA-functionalized Ag NPs were obtained according to the following procedure. 20 μL of 0.1 mM 4-MBA ethanol solution was added into 2 mL of Sample 2 and stirred at 30 °C for 12 h. at last, the above solution centrifugated at 8500 rpm for 15 min to get rid of the excess 4-MBA molecules and the remnant was redispersed in 2 mL of de-ionized water.

The preparation method of DTNB-functionalized Ag NPs was almost the same as that of 4-MBA-functionalized Ag NPs. The only difference was that 50 μL of 1 mM DTNB ethanol solution was added instead of the 4-MBA solution.

The other molecules with the thiol groups functionalized with Ag NPs were same as DTNB.

The preparation method of R6G-functionalized Ag NPs is the same as above. The difference was that 100 μL of 1 mM R6G ethanol solution was added.

4-MBA and DTNB co-functionalized Ag NPs were fabricated using the following method. 2 mL of Ag NPs solution was centrifugated at 8500 rpm for 15 min to remove the excess reagents. The precipitate was redispersed in 2 mL of de-ionized water, followed by the addition of 4-MBA ethanol solution (20 μL of 0.1 mM 4-MBA) during the stirring. After 2 h, the DTNB ethanol solution (200 μL of 0.1 mM DTNB) was added and the mixture was stirred for 10 h. finally the resultant solution was centrifuged at 8500 rpm for 15 min and the precipitate was redispersed in 2 mL of de-ionized water.

### Encapsulating the SERS-Ag NPs with silica

SERS reporters-functionalized Ag NPs were further encapsulated with a silica shell based on the Stöber’s method. As-prepared SERS-functionalized Ag NPs solution (2 mL) was mixed with isopropanol (8 mL) in a 15 mL plastic conical tube. Dimethylamine solution (0.2 mL, 20 wt.-%) was added to the mixed solution, followed by the addition of TEOS in isopropanol (0.2 mL, 10 mM) three times within 6 h (at a time interval of 2 h). After shaking for 10 h, The reaction mixture was then centrifuged at 8500 rpm for 15 min and the Ag@SiO_2_ NP precipitate was redispersed into ethanol for further washing. After three times of washing, Ag@SiO_2_ NPs were obtained and redispersed into deionized water or ethanol (2 mL) for characterization and further functionalization. Shell thickness of colloid/SiO_2_ particles can be simply controlled by the amount of TEOS.

### Synthesis of MUA-based SERS tags

The detailed procedure sees our before article^36^. The difference is only that the MUA-modified Ag NPs reacted with the Raman molecules, and then were coated with silica.

### Characterization

The size of NPs were measured by field emission scanning electron microscopy (FE-SEM, Hitachi, S-4800). UV-vis absorption spectra were obtained at room temperature using a UV-3100 spectrophotometer. The Raman spectra were measured at room temperature by an Ocean Optics QE6500 Raman spectrometer system equipped with a 785 nm laser.

## Additional Information

**How to cite this article**: Zhang, Y. *et al.* A facile and general route to synthesize silica-coated SERS tags with the enhanced signal intensity. *Sci. Rep.* **5**, 14934; doi: 10.1038/srep14934 (2015).

## Supplementary Material

Supplementary Information
