# Supplementary material for: A facile and general route to synthesize silica-coated SERS tags with the enhanced signal intensity
Source: Sci Rep. 2015 Oct 9;5:14934. doi: 10.1038/srep14934 (PMC4598865; doi:10.1038/srep14934)
Supplement: Supplementary Information [file srep14934-s1.doc]

A facile and general route to synthesize silica-coated SERS tags with the enhanced signal Intensity

Youlin Zhang,^+^ Xiaokun Li,^+^ Bin Xue, Xianggui Kong,^*^ Xiaomin Liu, Langping Tu, Yulei Chang

**Supporting information**

**Table S1.** The influence of the dielectric constant and the concentration of the salt on the stability of Ag NPs in alcoholic solutions according to the Figure 1.

| Sample | Water/isopropanol | *ε* | Concentration of salt (mM) | *V*_elec_ |
| --- | --- | --- | --- | --- |
| 1 | 1:0 | 80 | 7 | >10k_B_T |
|  | 1:1 | 30 | 7 | <10k_B_T |
| 2 | 1:1 | 30 | 7×10^-2^ | >10k_B_T |
|  | 1:4 | 21 | 7×10^-2^ | <10k_B_T |
| 3 | 1:4 | 21 | 7×10^-4^ | >10k_B_T |
|  | 0:1 | 18 | 7×10^-4^ | >10k_B_T |


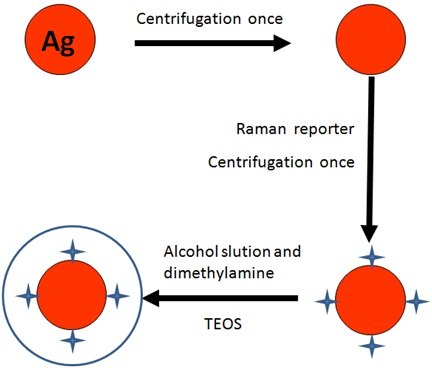


**Scheme S1.** Illustration of the preparation of the silica-coated core-shell SERS tags


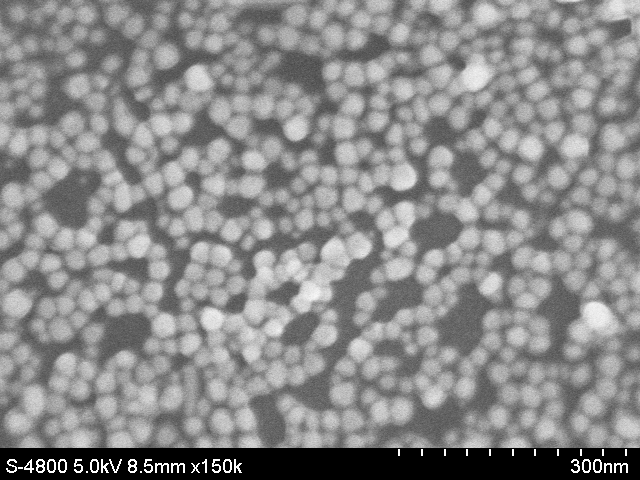


**Figure S1.** FE-SEM image of citrate-stabilized Ag NPs.


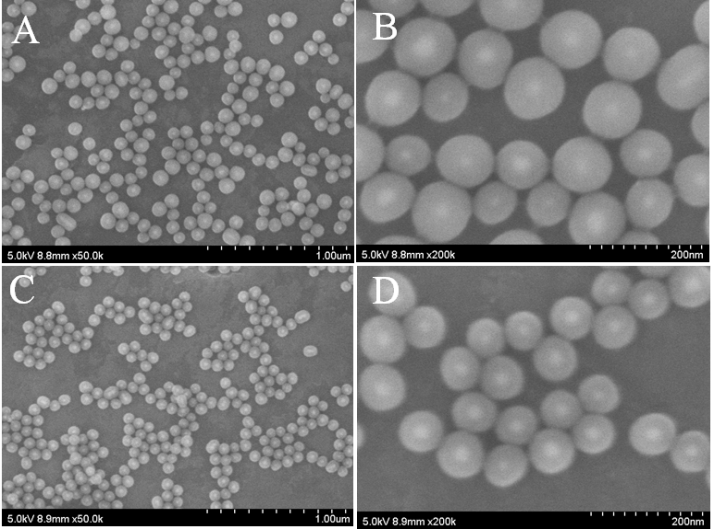


**Figure S2.** FE-SEM of silica-coated Ag NPs (the diameter of the Ag NPs is 25 nm) produced by altering the amount of TEOS added. The shell thicknesses are about 40 nm (A), and 25 nm (C); (B) and (D) are the corresponding higher magnification ratio.

**Encapsulation of Ag NPs with silica**

The above results demonstrate that the Ag NPs can be stable in alcoholic solution by removal of the residual salt. In the following, we shall prove that the Stöber method can produce a silica shell, using TEOS, on the citrate-stabilized Ag NPs. This method involves base-catalyzed hydrolysis of TEOS to generate silica sols, followed by nucleation and condensation of these sols on the surfaces of Ag NPs. In our experiment, dimethylamine was used as an alternative to ammonia to catalyze silica shell growth on Ag NPs, since ammonia can etch Ag NPs.^1^ It is known that the thickness of the silica shell can be accurately controlled by precisely adding the amount of TEOS.^2^ Here, we controlled the thickness of silica shell by repeatedly adding the TEOS into the reacting solution at a time interval of 2 h. Monodisperse Ag@SiO_2_ NPs were successfully prepared via our modified Stöber method. The morphologies of the Ag and Ag@SiO_2_ were characterized with FE-SEM. The synthesized Ag NPs were nearly spherical with an average diameter of 25 nm (Figure


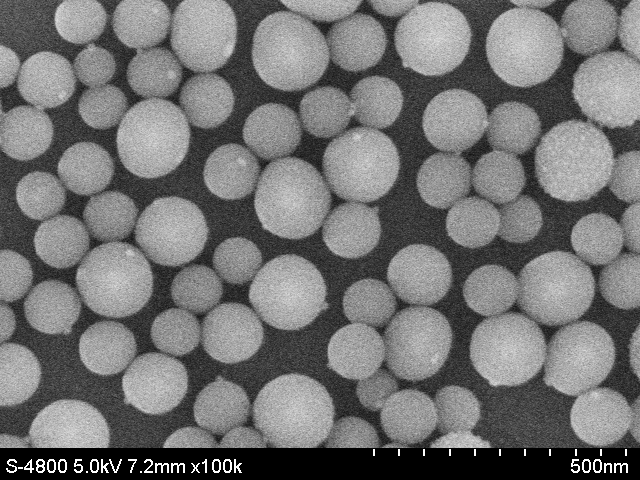

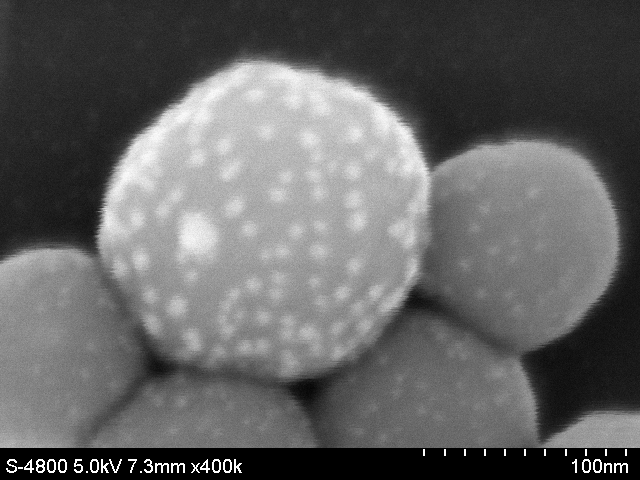


**Figure S3.** FE-SEM images for the SiO_2_-coated citrate-stabilized Ag NPs with ammonia as catalysis with different magnification.

S1). The Ag@SiO_2_ NPs were prepared upon addition of TEOS with different amount relative to citrate-stabilized Ag NPs (Figure S2A-D). As is shown in the figure, the Ag@SiO_2_ NPs are nearly spherical and uniform, and their diameter increases with increasing TEOS amount. For both samples, no secondary nucleation of small silica colloids is observed, confirming the sufficient high total particle surface per volume. The core/shell nanostructures are clearly seen due to the strong contrast between the white cores and gray shells, which provides direct evidence that the Ag NPs are completely encapsulated with silica shells. The thickness of the silica shells can be tuned by varying the amount of added TEOS. Ammonia has been often used as catalyst in this sort of synthesis and its etching of Ag NPs have also been reported.^1^ In this work it was also carefully studied. Figure S3 shows the image of Ag@SiO_2_ using ammonia as catalyst. The Ag NPs with the diameter of 25 nm was etched into the diameter of about 5 nm, adsorbed on the surface of silica NPs. Therefore ammonia is not appropriate for catalysis in this work.

**The enhancement factors of 7 kinds of Raman molecules**

Measurements of Raman cross sections of 7 kinds of Raman molecules on Ag NPs are complicated by the fact that its surface coverage is not exactly known and might vary depending on the Raman molecules. Therefore, we consider analytical enhancement factors (AEF) as defined by the reference (3) and used extensively in SERS research: AEF=(*I*_SERS_/*c*_SERS_)/(*I*_ref_/*c*_ref_), where *I* is the Raman intensity and *c* represents the concentration of the analyte. The calculated AEF are 1.5×10^7^, 2×10^5^, 8×10^3^, 1.8×10^5^, 2.1×10^6^, 3.6×10^4^ and 1.1×10^5^ for 4-MBA, DTNB, R6G, 2-MBA, ATP, 4-FTP and 4-AMTP, respectively.

**The influence of silica shell on the absorption spectra of Ag NPs**

The Ag NPs absorption maximum located around 406 nm. After ligand exchange with 4MBA, the absorption shifted to the red by 3 nm. The thickness of silica shells was varied with ranging from 20 nm to 60 nm. Compared to the peak of Ag@4MBA, the absorption maximum shifted to the red from 409 nm to 426 nm, resulting from the increase in local refractive index around the particles. When the silica shell thickness changed from 20 nm to 60 nm, scattering became significant.

**Figure S4**. The influence of shell thickness on the absorption spectra of Ag NPs encapsulated with silica shell. The silica shell thicknesses are 20 nm, 44 nm and 62 nm.

**Simulation of electric field of Ag@SiO_2_ core-shell structure**


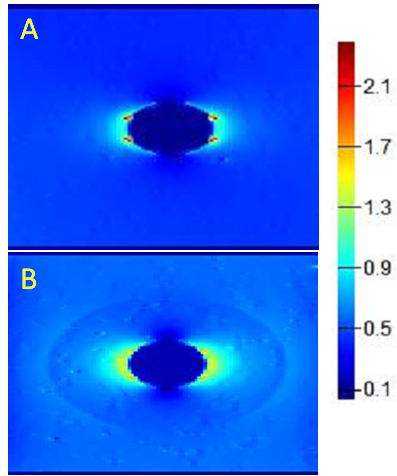


**Figure S5.** The FDTD calculated local electric field of Ag NPs (A) and Ag@SiO_2_ NPs (B) on the XY plane at the excitation wavelength of 785 nm.

A thorough investigation of the optical properties of Ag and Ag@4MBA@SiO_2_ is crucial to understand their SERS properties. The experimental extinction spectra (Figure S4) show a strong dependence on the encapsulation, which can be attributed to a relatively high refractive index of the SiO_2_ shell. Because the Ag NPs are monodispersed, there is no inter-particle coupling between the Ag NPs in our sample. The electric field enhancement should have a similar spectral dependence as the extinction spectrum. This has been confirmed by the simulated local electric fields of Ag and Ag@SiO_2_ NPs.

Numerical simulations were carried out through a finite difference time domain (FDTD) method (Lumerical Solutions, Inc.). The incident beam was a total field-scattered field (TFSF) source of light with its propagation directions along the Z-direction. The amplitude of the electric field of the incident light was taken to be 1.0 V/m. The whole simulation region was assumed in water. We used perfectly matched layer absorbing boundary conditions. We used the Palik’s bulk dielectric data for silver and SiO_2_.

**Figure S6.** Raman spectra obtained silica-coated R6G Ag NPs before (A) and after (B) the addition of 4-MBA (10×10^-6^ mol/L)


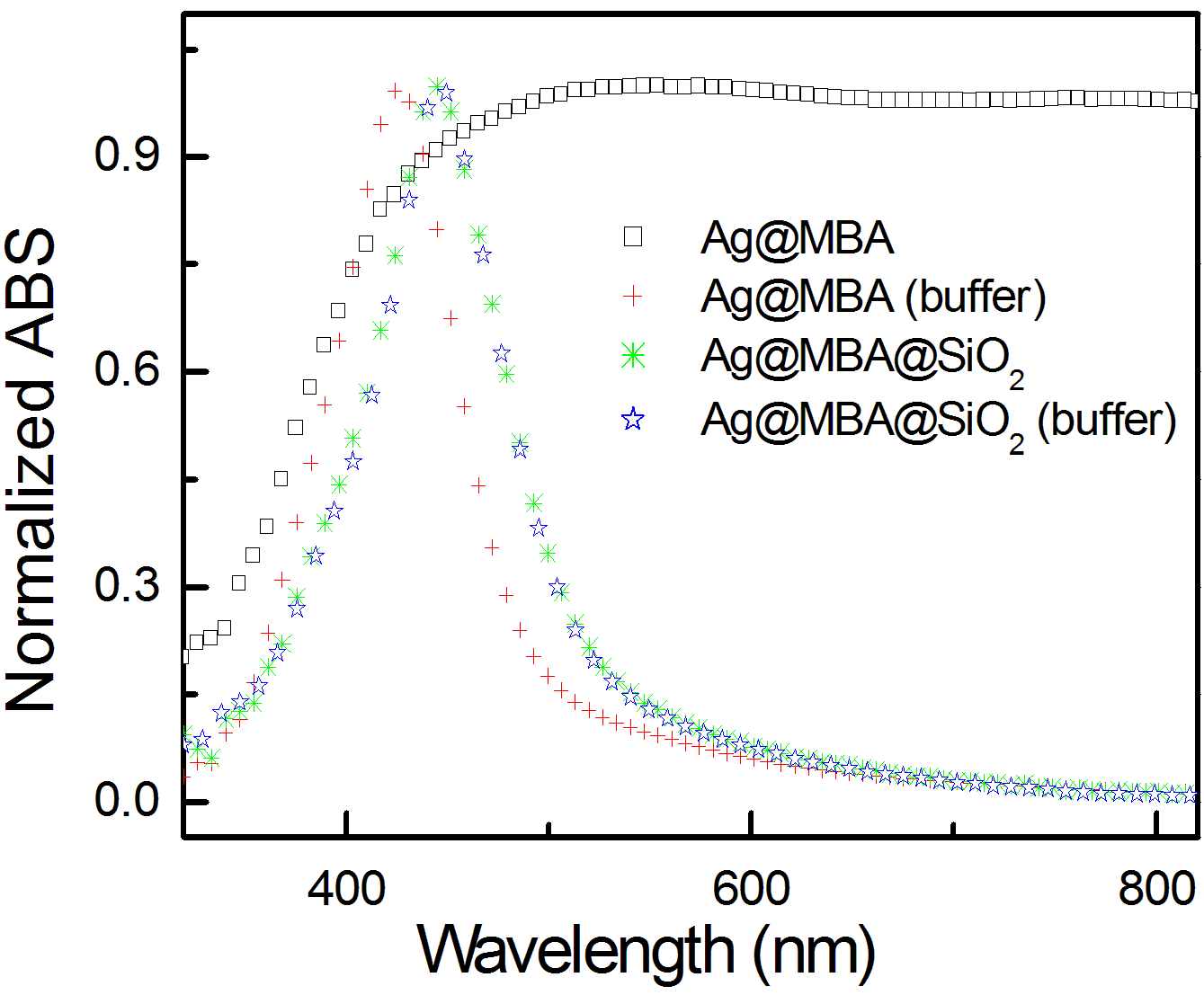


**Figure S7.** UV-vis spectra of Ag and Ag@SiO_2_ Raman tags before and after exposure to buffer solutions containing 100 mM NaCl.

**The stability of silica-coated SERS tags**

In the presence of NaCl, the color of the Ag@SiO_2_ remained almost unchanged, while in the case of Ag NPs the final solution gradually turned colorless because the salts gave rise to the aggregation of the Ag NPs followed by precipitating at the bottom. The spectra of Ag@SiO_2_ colloids do not show significant difference in the absence or presence of salts, in contrast to Ag NPs where a reduced absorbance and a significant red shift of the absorption band occur due to aggregation of the Ag NPs. Furthermore, the coated particles were stable in organic solvents such as methanol and acetone that are known to precipitate protein-stabilized Ag NPs. They were extremely stable in aqueous solution and original SERS activity remained and no aggregation even after 12 months storage.

**References**

1. C. Xue, X. Chen, S. J. Hurst and C. A. Mirkin, *Adv. Mater.,* 2007, **19**, 4071-4074.
2. C. Graf, D. L. J. Vossen, A. Imhof, A. van Blaaderen, *Langmuir,* 2003, **19**, 6693-6700.
3. E. C. Le Ru, E. Blackie, M. Meyer, and P. G. Etchegoin, *J. Phys. Chem. C,* 2007, **111**, 13794-13803.
